# Should Physicians Be Permitted to Refuse Follow-Up Care to Patients Who Have Received an Organ Transplant Through Organ Trafficking?

**DOI:** 10.3389/ti.2023.11529

**Published:** 2023-10-06

**Authors:** Yoshiyuki Takimoto

**Affiliations:** Department of Biomedical Ethics, Faculty of Medicine, The University of Tokyo, Tokyo, Japan

**Keywords:** transplant tourism, ethics, follow-up care, organ transplant, organ trafficking

## Abstract

In 2018, the Istanbul Declaration stated that organ transplantation via organ trafficking is a crime. Since then, the number of medical institutions in Japan who refuse follow-up care to patients who have undergone unethical organ transplantation overseas has been gradually increasing. Deterring transplant tourism involving organ trafficking is an issue that must be addressed by the government, medical institutions, and individual physicians. The refusal of medical institutions and individual physicians to provide follow-up care after organ transplantation may challenge the idea of the incompatibility thesis; moreover, it may be ethically justified in the context of conscientious objection if it is based on the belief of deterring transplant tourism instead of punitive motives or a reluctance to support a criminal activity. However, conscientious objection based on a belief in fair transplantation care is conditional; according to the compromise approach, it is limited to particular conditions, such as that the patient’s medical state does not require urgent care and that the patient is reasonably able to receive follow-up care at another institution.

## Introduction

Transplant tourism is a major social and ethical issue concerning organ transplant medicine in Japan.

The Declaration of Istanbul on Organ Trafficking and Transplant Tourism states that travel for transplantation is considered transplant tourism if “it involves organ trafficking and/or transplant commercialism or if the resources (organs, professionals, and transplant centers) devoted to providing transplants to patients from outside a country undermine the country’s ability to provide transplant services for its own population.” Transplant tourism is unethical because it violates ethical principles of justice and fairness and undermines human dignity [1].

An issue closely related to transplant tourism is organ trafficking and the removal of organs from executed prisoners. Organ trafficking is defined in the 2018 edition of the Istanbul Declaration [2] as any of the following: a) removing organs from living or deceased donors without valid consent or authorization or in exchange for financial gain or comparable advantage to the donor and/or a third person; b) any transportation, manipulation, transplantation or other use of such organs; c) offering any undue advantage to, or requesting the same by a healthcare professional, public official, or employee of a private sector entity to facilitate or perform such removal or use; d) soliciting or recruiting donors or recipients, where carried out for financial gain or comparable advantage; or e) attempting to commit, or aiding or abetting the commission of, any of these acts. The Istanbul Declaration states that organ trafficking should be criminalized [2]. The removal of organs from executed prisoners was previously legal in China, but has been prohibited since 2015. This practice can be considered unethical due to certainty of the individual’s consent. In this regard, it may also constitute organ trafficking [3].

The Declaration of Istanbul states that medical professionals should help prevent transplant tourism and organ trafficking activities [4].

Owing to the unique Japanese view of life and death influenced by Shintoism and Buddhism, organ transplants from brain-dead donors is rare in Japan. Consequently, transplant tourism emerges as a means of acquiring organs through procedures that exclude living donors, including heart transplants. Recently, there has been a rise in the practice of transplant tourism, not only in cases of organ transplantation that necessitate a brain-dead donor, but also in kidney and liver transplantation, which allow for living donor transplantation. In transplant tourism, organs are obtained by placing Japanese patients at the top of waiting lists or engaging in organ trafficking. As transplant tourism most frequently occurs when potential recipients travel to countries where laws prohibiting organ trafficking are riddled with loopholes or poorly enforced, transplant tourism often exposes individuals to the risk of encountering organ trafficking [5].

To mitigate the risks of exposure to organ trafficking efforts are needed both at the individual level—for example, transplant specialists should increase patient awareness and education—and at the national level—such as excluding follow-up care from public healthcare insurance coverage and increasing transplants from brain-dead donors. Following the declaration, several hospitals in Japan announced that they would not provide follow-up care to patients suspected of participating in organ trafficking.

Apropos of this, a university hospital refused to provide follow-up care to a transplant tourism patient for similar reasons, which resulted in the patient filing for damages. The hospital had a policy of not examining or treating patients who had received kidney transplants in China involving organ trafficking (via organ brokers). Upon noting insufficient information in the patient’s letter of referral regarding the course of treatment and other details, the doctor refused to treat the patient [6]. The court found the purpose and objective of the rule legitimate and the reasons satisfactory for the doctor’s decision. The court stated, “It is effective and reasonable to try to curb organ trafficking and transplant tourism indirectly by means such as denying treatment to patients who have undergone such organ transplantation.” However, the court did not justify the refusal of medical treatment based solely on the existence of the rule; rather, it made a judgment after a comprehensive consideration of the patient’s urgent need for medical treatment, the possibility of the case being handled at another medical institution, the purpose of refusing the medical treatment, and the justifiability of the refusal. Furthermore, the director of a non-profit organization was arrested in February 2023 on suspicion of mediating unauthorized organ transplants overseas. Consequently, the Ministry of Health, Labour and Welfare indicated its plan to conduct a survey in 2023 with patients who visited medical institutions in Japan after their transplants. Thus, in Japan, medical institutions’ attempt to prevent organ trafficking by refusing follow-up medical care to patients suspected of illegal organ transplant is becoming popular; however, these actions are causing social problems due to insufficient discussion.

The practical issue of how to confirm transplant tourism involving organ trafficking cannot be ignored. The Declaration of the Istanbul Custodian Group recommends that to specifically identify patients who have undergone transplants via organ trafficking, physicians should be provided with guidance and training so that they can identify the circumstances consistent with organ trafficking [6]. Examples of such circumstances include a transplant patient who received a transplant abroad without having been referred to do so by their treating physician or team, absent or incomplete information on the relationship between the recipient and the donor, absent or incomplete information on the patient’s clinical course, absence of detailed medical records from both the donor and recipient, and immediately seeking care at a hospital or emergency room [7].

Furthermore, assuming that these practical issues are cleared, there is still an ethical debate as to whether it is acceptable to refuse follow-up care to a patient who is certain to have undergone a travel transplant involving organ trafficking.

## Aims

This viewpoint examines whether the refusal of follow-up care for transplant tourism patients who received a transplant via organ trafficking is ethically acceptable, using two prevailing rationales—deterrent effect and conscientious objection.

### An Ethical Analysis of the Reasons for Physicians’ Reluctance to Provide Follow-Up Care to Transplant Patients Involving Organ Trafficking

Some physicians are reluctant to treat patients who have received unethical and illegal organ transplants abroad [8]. Some of the reasons for this are described next.

#### Breach of Trust in the Physician–Patient Relationship

The first reason for this could be the difficulty in providing follow-up care owing to the breach of trust in the physician–patient relationship [9]; the patient may have participated in transplant tourism against the physician’s recommendations. When the physician-patient trust is lost, it becomes difficult to provide effective treatment. Consequently, the physician would be ethically exempted from providing treatment to the patient because they would not be able to fulfill the duty of beneficence. However, if a patient undergoes an organ transplant via organ trafficking, the physician-patient relationship may rarely be so broken that the physician cannot provide effective follow-up medical care because of the physician’s distrust toward the patient and *vice versa*.

#### Reluctance to Provide Medical Care to Criminals

The second possible reason is psychological resistance to providing treatment to individuals who have committed the criminal act of transplantation via organ trafficking. There may be feelings of discrimination against these individuals, which underlie physicians’ concerns about the ethics of providing medical care to criminals [10]. However, the appropriateness of medical care must be judged purely based on medical indications, rather than the patient’s attributes. A physician is expected to provide uniform medical care to all patients with the same condition, thus fulfilling the principle of fairness. As stated in the Hippocratic oath, the fairness of providing medical care regardless of individual attributes has been a professional ethic for physicians since ancient times [11]. Therefore, denying follow-up care to a patient who has received an illegal organ transplant simply because they are a criminal is unsupportable.

#### Reluctance to Be Involved in Criminal Activity

The third reason may relate to personal beliefs: follow-up care is ethically unacceptable because it supports organ transplantation via organ trafficking, which is a criminal act; therefore, a physician’s medical practice would indirectly contribute to criminal activity. However, this belief is ethically denied owing to the principle of the double effect that postulates that the provision of follow-up care is not complicit in organ trafficking, rather that it is intended to provide physical management of the immediate post-transplant patient [12].

#### Realization of Fair Organ Transplantation

The fourth reason may be related to professional obligation: doctors have a duty to serve public interest, and to realize fair organ transplantation, it would be better for them to refuse to provide medical care after an illegal organ transplant. Would the fourth reason for deterring unethical transplant tourism ethically justify the refusal of follow-up care? The principle of fairness encourages the fair allocation of medical resources. Organ trafficking and transplant tourism are exploitative, as they obtain organs from citizens of poor countries and provide them to those in rich countries. This creates an ethical concern about fairness because patients in need of organ transplants in poor countries are denied the treatment opportunity. Moreover, efforts to prevent organ trade and transplant tourism appear to be an ethical obligation for physicians and medical institutions in accordance with the principle of fairness.

#### Conflict Between Principle for Justice and Beneficence

However, the principle of beneficence conflicts with the principle of fairness concerning follow-up care. The principle of beneficence calls on physicians to not only avoid harm but also benefit patients and promote their welfare [13]. Physicians are obligated to provide medically beneficial follow-up care. The refusal to provide such care results in the patient losing the treatment opportunity, especially in the absence of other medical facilities in the vicinity [14]. The probability of this situation is high because, unlike abortion and other procedures, only few medical institutions can provide follow-up care after a transplant. Refusal to provide follow-up care can result in serious medical and social risks for the patient. If proper follow-up care is lacking, there is a risk of loss of function in the transplanted organ. For example, if the patient is a post-kidney transplant patient, they may develop end-stage renal failure. There is also the risk of a shorter life expectancy. Lack of proper follow-up can result in, for example, post-kidney transplant patients requiring dialysis, intensive care due to infection, or re-inclusion on the transplant waiting list. This imposes a burden on the society because it requires medical expenses and resources that could otherwise be spared.

#### Certainty of Deterrence

Additionally, there is an issue regarding certainty in terms of deterrence, which is supported by the principle of fairness. It is unclear how effective refusal would be as a deterrent, and there is no certainty that the duty of fairness would be fulfilled. Thus, regarding certainty, the duty of beneficence (providing follow-up care to the patient) is more important for balancing the conflict between the two duties. This argument about certainty in sacrificing individual interests for the good of the community is also recognized as an important ethical concern in bedside rationing, where there is no assurance that the resources saved by not providing medical care to a certain patient will be utilized more efficiently for the benefit of other patients [15]. If the emphasis is on the consequence of achieving a fair allocation of medical resources (fair organ transplantation) as required by the principles of fairness, it would be difficult to ethically justify the denial of follow-up care based on the same principle. This is because the achievement of fair organ transplantation remains uncertain even after denying follow-up care.

### Ethical Analysis of Conscientious Refusal to Provide Follow-Up Care to Transplant Patients Involving Organ Trafficking

This raises the question of whether it is always unacceptable for a physician to place the duty of fairness above that of beneficence. The above discussion focuses on the consequences of whether or not fair organ transplantation will be achieved. Conversely, it is also possible to focus on the intention of physicians to realize fair organ transplantation.

#### Conscientious Objection to Medical Treatment

The obligation to provide medical care can be divided into legal and ethical obligations. In terms of legal obligations, a physician is not considered obligated to provide medical care to a patient unless it is an emergency situation. In terms of ethical obligation, known as the principle of beneficence, a physician is considered obligated to provide medical care if doing so would contribute to the medical benefit of the patient. A physician’s refusal to provide ethical obligatory medical care can be understood based on the concept of conscientious objection, which means refusing a duty based on one’s religious, ethical, or political beliefs [16]. In medicine, conscientious objection is sometimes recognized as “the right to refuse performing of a medical procedure for one’s own beliefs, even if one is obligated to do so” [17]. A representative example is the inclusion of a conscientious objection clause in almost all abortion legislations worldwide. This clause grants medical providers with certain religious beliefs, such as Christian beliefs, the right to refuse to perform an abortion (if they consider it a sin). This is interpreted as follows: just as a woman has the right to self-determination regarding abortion, a medical professional has the right to do what they believe is ethically correct.

#### Conscientious Objection to Follow-Up Medical Care After Organ Transplantation

Follow-up care after organ transplantation is a legitimate medical practice and no physician would object to its importance. The difference between this specific type of follow-up care and common medical practices is that the former is subject to conscientious objection for physicians for two reasons.

##### Conscientious Objection to Being Involved in Criminal Activity

First, underlying the conscientious objection of physicians to provide follow-up care after transplantation of trafficked organs, may be the belief that they should not be complicit in criminal medical care. Physicians may not be resistant to the concept of follow-up care itself, but to their involuntary participation in a criminal procedure. In this case, even if it is legal to provide follow-up care, the physician could experience an ethical resistance, which is considered conscientious objection. However, this belief is ethically challenged by the principle of double effect [12]. The act of providing follow-up care is neutral in value. The physician’s intention is to ensure the ongoing medical health of the patient following an organ transplant, and not to knowingly participate in any criminal activity related to transplantation via organ trafficking. Providing follow-up care has positive medical outcomes for the patient, regardless of whether it leads to complicity in criminal activity.

##### Conscientious Objection to Deter Transplant Tourism

The most common and prevailing conscientious objection of follow-up care in this case may be the belief that to deter organ transplantation, no follow-up care should be provided for those involved in the criminal act of organ trafficking.

###### Certainty of Effectiveness

The first problem with this belief is uncertainty about if refusing will promote fairness in organ transplants. However, for conscientious objection, the motivation behind beliefs is more important than the consequences. Therefore, the validity of the belief is more important than the uncertainty about the deterrent effect of refusing follow-up care in case of unfair organ transplantation.

###### Which Fairness Should Be Prioritized?

The second problem is the conflict within the principle of fairness–between the ethical imperatives to provide the same treatment to patients with the same condition and to create fair medical resources by conscientious objection to follow-up care [18]. To justify a physician’s belief that their duty of fairness to society takes precedence over their duty of fairness to the patient, it would be necessary for that patient to have reasonable access to follow-up care from another physician, as is required in the “compromise approach” of conscientious objection [18].

###### Incompatibility Thesis

The third problem pertains to the “incompatibility thesis,” which states, “the duties of a healthcare professional are incompatible with the demands of conscientious objection” [19]. From the standpoint of the “incompatibility thesis,” healthcare professionals should always provide legitimate, safe, and (from the patient’s perspective) beneficial treatment, regardless of the moral and personal values of the individual. Wicclair [16] suggests that for a conscientious objection to be recognized, the core ethical value on which the objection is based should be consistent with one or more core values in medicine. From this perspective, conscientious objection to follow-up care differs from the conscientious objection to abortion or assisted suicide based on the physician’s personal values because it is based on the core medical ethical value of fairness [20]. In some cases, it may be considered acceptable to challenge the “incompatibility thesis” and give precedence to the fairness of healthcare over the wellbeing of the patient.

###### Impact on Patients From Disadvantaged Backgrounds

Another problem with conscientious objection is that it can be particularly harmful to individuals from disadvantaged backgrounds, including those with lower socioeconomic status, rural or remote residents, and individuals with poor health literacy. However, considering the enormous cost of transplantation tourism, overlooking the problem could lead to greater socioeconomic disparities both nationally and internationally.

### Acceptable Ethical Conditions for Conscientious Objection to Follow-Up for the Purpose of Deterring Transplant Tourism Involving Organ Trafficking

Accordingly, refusing follow-up care based on the belief that it will deter organ trafficking can be recognized as a conscientious objection, which overcomes several problems. However, the objection is conditional. First, for the duty of fairness to take precedence over the duty of beneficence, the demand of beneficence should not be strong. If the patient’s condition requires urgent follow-up care, the call for beneficence is considerably strong, and the conscientious objection to providing follow-up care is not ethically justified [21]. Second, when conscientious objection takes precedence over the duty of beneficence, the “compromise approach” should be followed, as conscientious objection is not an unconditionally recognized physician right [22]. In the “compromise approach,” the conscientious objection to providing legitimate goods and services within the practitioner’s capacity is considered compatible with the professional’s duty if it does not unduly interfere with the patient’s timely or convenient use of the goods and services [18]. According to this approach, a conscientious objection to follow-up care must be based on a situation in which follow-up care is relatively easy to obtain from other medical facilities. In cases requiring advanced medical care, with few alternative medical institutions or unavailability of follow-up care at nearby medical institutions, conscientious objection is not acceptable.

### Specific Acceptable Conditions for Conscientious Objection to Follow-Up for the Purpose of Deterring Transplant Tourism Involving Organ Trafficking

Thus, for a conscientious objection to be ethically acceptable, it must be based on a professional obligation to achieve fairness in organ transplantation care, not merely on a personal belief, or the fact that the patient’s condition is not urgent and follow-up care from other medical facilities is available. Specifically, conscientious objection to deter organ trafficking for realizing fair organ transplantation may be acceptable if the following conditions are met:

(i) It is clear (or strongly suspected) that the person was involved in illegal organ trafficking.
(ii) The situation is not medically urgent.
(iii) It is possible for the patient to receive follow-up medical treatment at another medical institution.
(iv) If medical treatment has already started, follow-up treatment should be provided until the case is referred to another physician.
(v) The possibility of refusing follow-up care must be presented in advance.

The Canadian Society of Nephrology [23] states that while it does not intend to promote refusal to provide follow-up care to patients, individual physicians may choose to delegate follow-up care to another professional in non-urgent situations.

The Declaration of the Istanbul Custodian Group has recommended that with regard to the follow-up of travel transplant patients, the primary duty of healthcare professionals in any circumstance is to ensure the provision of care, and that it is not the responsibility of the healthcare professional to sanction patients suspected of criminal activity [7]. Therefore, it states that post-transplant tourism patients should be promptly referred for evaluation at a transplant center to ensure proper screening and care, particularly in managing infectious diseases. It also recommends applying this principle to patients who have received transplants through organ trafficking. Moreover, it emphasizes that medical institutions and public insurance should not cover the cost of organ trafficking-related transplants, but follow-up care after a transplant via organ trafficking should be paid for in the same manner as other transplant patients, provided that relevant information is recorded in an official transplant registry. While advocating these actions, the report acknowledges that in non-emergent situations, individual physicians may choose to defer the follow-up care of these patients to another physician.

Stating the policy in advance is considered important for ensuring procedural justice amidst the controversy surrounding conscientious objection [24]. Additionally, when making a conscientious objection, it is necessary to provide reasons for the refusal rather than merely stating the refusal [25]. Thus, if a physician refuses follow-up care based on their conscience regarding fair organ transplantation care, it is necessary to explain this to the patient in advance. Furthermore, practically, it would be useful for medical institutions to present their policy beforehand to avoid problems with patients, ensuring smooth access to medical care so that patients can avoid institutions that may refuse follow-up care.

However, certain practical difficulties remain, such as the definition of “another medical institution,” criteria for determining “urgency,” and how the involvement in “organ trafficking” can be confirmed. If no medical institution is available for follow-up care within the patient’s residential prefecture or the likelihood of a life-threatening condition is high, follow-up care should be provided.

## Conclusion

The principles of transparency and continuity of care that apply to patients who receive an organ domestically should also apply to transplant tourism patients who received a transplant via organ trafficking. According to the concept of conscientious objection, in non-emergent situations, individual physicians may elect to defer the care of these patients to another physician. However, there are numerous requirements to satisfy this condition, such as determining the illegitimacy of transplant tourism due to its involvement in organ trafficking and assuring proper follow-up at another medical institution.

Thus, concerning refusal to provide follow-up care to a patient who underwent an unethical organ transplant, the appropriate attitude for a physician is “when in doubt, do what is in the patient’s best interest.”

## Data Availability

The original contributions presented in the study are included in the article/supplementary material, further inquiries can be directed to the corresponding author.
